# A comprehensive phylogeny and revised taxonomy of Diadectomorpha with a discussion on the origin of tetrapod herbivory

**DOI:** 10.1098/rsos.231566

**Published:** 2024-06-26

**Authors:** Jasper Ponstein, Mark J. MacDougall, Jörg Fröbisch

**Affiliations:** ^1^ Humboldt-Universität zu Berlin, Unter den Linden 6, 10117 Berlin, Germany; ^2^ Museum für Naturkunde Berlin, Invalidenstraße 43, 10115 Berlin, Germany; ^3^ Oertijdmuseum, Bosscheweg 80, 5283 WB Boxtel, The Netherlands

**Keywords:** Diadectomorpha, tetrapoda, phylogeny, mandibular anatomy, herbivory, Palaeozoic

## Abstract

Among terrestrial tetrapods, the origin of herbivory marked a key evolutionary event that allowed for the evolution of modern terrestrial ecosystems. A 100 Ma gap separates the oldest terrestrial tetrapods and the first undisputed herbivorous tetrapods. While four clades of early tetrapod herbivores are undisputed amniotes, the phylogenetic position of Diadectomorpha with respect to Amniota has long been controversial. Given that the origin of herbivory coincides with the oldest amniotes, and obligate herbivory is unknown within amphibians, this suggests that a key adaptation necessary to evolve obligate herbivory is unique to amniotes. Historically, phylogenetic analyses have found Diadectomorpha as the sister-group to amniotes, but recent analyses recover Diadectomorpha as sister-group to Synapsida, within Amniota. We tested whether diadectomorphs are amniotes by updating the most recent character–taxon matrix. Specifically, we added new characters from the lower jaw and added diadectomorph taxa, resulting in a dataset of 341 characters and 61 operational taxonomic units. We updated the description of five diadectomorph jaws using microcomputed tomography data. Our majority-rule consensus places Diadectomorpha as sister-group to Synapsida; other methods do not recover this relationship. We revise diadectomorph taxonomy, erecting a new species from the early Permian Bromacker locality, Germany, and a new genus to accommodate ‘*Diadectes’ sanmiguelensis*.

## 1. Background

The evolution of herbivory in terrestrial tetrapods, here defined as a feeding strategy in which the bulk of the nutrients are derived from the breakdown of cellulose (*sensu* [1] marked one of the key innovations leading to the development of modern ecosystems). Following the initial evolution of tetrapod herbivory, five tetrapod clades independently acquired adaptations towards herbivory in rapid succession over the course of the Late Carboniferous to early Permian. These clades are the diadectomorph clade Diadectidae, the synapsid clades Edaphosauridae and Caseidae, the parareptilian clade Bolosauridae and the eureptilian clade Captorhinidae [2–5]. Typical adaptions to infer herbivory in fossil tetrapods include a robust, deep lower jaw with a dentition specialized in cropping and grinding and a barrel-shaped torso to host an enlarged digestive tract [2–4,6].

A significant time gap (*ca* 100 Ma) is present between the first occurrence of non-marine tetrapods in the Middle Devonian [7–9] and the oldest herbivorous tetrapods in the Late Carboniferous [2,4,5,10]. This first radiation of herbivorous tetrapods coincided with the origin of Amniota [10]. Until the middle Permian, herbivorous tetrapods remained a rare faunal component in their respective ecosystems [2,4,11,12]. Modern terrestrial community structures, which are dominated in absolute numbers by herbivores, do not become widespread globally until the middle–late Permian [13].

Herbivory has evolved repeatedly in each major amniote clade, including Synapsida [14–16], Lepidosauria [17–20], Archosauria [21–26] and Testudinata [27–29]. By contrast, the paucity of adaptations to herbivory in the other major clade of extant tetrapods, Lissamphibia, is apparent. The highly derived caudatan clade Sirenidae includes species that show adaptations towards omnivory [30] and few extant anurans supplement their diet with fruits or leaves [31,32]. Nevertheless, adaptations to obligate herbivory are not known within the members of the total-group Lissamphibia. The timing of the origin of herbivory coinciding with the origin of Amniota, as well as the skewed distribution of herbivory within tetrapods, raises the question whether a key biological innovation unique to Amniota was required to adopt herbivory [10]. By mapping the distribution of herbivorous taxa around the base of amniotes, one can narrow down when such an innovation likely first appeared.

In this context, Diadectomorpha is an interesting clade to study. While the other four clades of earliest herbivorous tetrapods are classified as amniotes [33], the placement of Diadectomorpha with respect to Amniota is disputed. Phylogenetic analyses traditionally place Diadectomorpha as the sister-group of Amniota [33–36], but this view has been challenged by recent studies involving endocranial characters that place diadectomorphs as early-branching synapsids [37–39].

The fossil record of Diadectomorpha is notable, spanning from the latest Carboniferous to early Permian from predominantly North America and Europe [40] and with numerous tracks attributed to diadectomorphs from Europe and northern Africa [41–43]. The clade includes the oldest known undisputed herbivorous tetrapod *Desmatodon hesperis* from the Upper Carboniferous Sangre de Cristo Formation of Colorado, USA [44]. Moreover, well-preserved crania and postcrania are known from the non-herbivorous Limnoscelidae [45–47] and Tseajaiidae [48–50]. The remaining diadectomorphs, or all diadectomorphs more closely related to *Diadectes* than to *Tseajaia campi*, are classified as diadectids (cf. [40]). Throughout their evolutionary history, diadectids gradually improved adaptions to a herbivorous diet, such as a deeper lower jaw and labiolingual expansion of the molariform cheek teeth [40].

**Figure 1.**
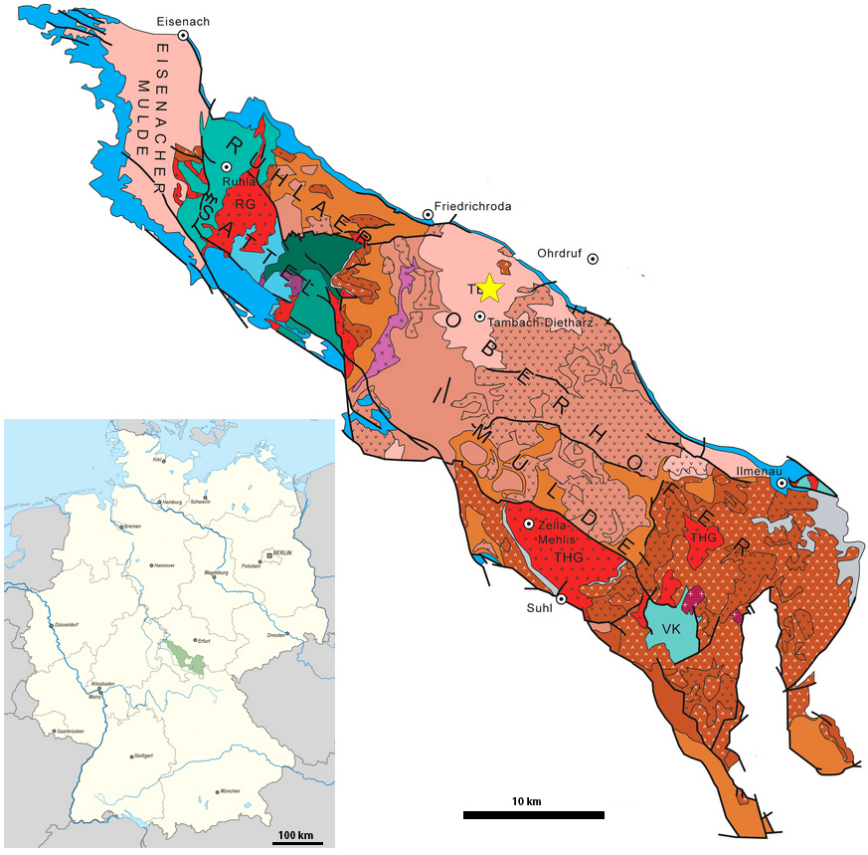
Location of the Bromacker locality (yellow star) within the Thuringian Forest in Germany, modified from Lützner *et al.* [51]*.* The location of the Thuringian Forest within Germany is indicated in green—the Thuringian Forest basin corresponds to the western part of the Thuringian Forest.

When present, diadectids, like the other early herbivorous tetrapods, often represent a minor component of their respective tetrapod fauna. This overall pattern, however, is notably different at the Bromacker locality from the early Permian of Thuringia, Germany (figure 1), which preserves the earliest herbivore-dominated fauna [52–55]. Four herbivorous tetrapod taxa are currently recognized—the diadectids *Diadectes absitus* and *Orobates pabsti*, the bolosaurid *Eudibamus cursoris* and the caseid *Martensius bromackerensis* [56–60], with skeletal elements of diadectids being most common [53]. Berman *et al*. described both the external cranial and postcranial anatomy of *D. absitus* [59] and *O. pabsti* [56], while Klembara *et al.* [61] more recently reconstructed parts of the internal and external cranial anatomy of *D. absitus*. The mandible and the palatal region of the Bromacker diadectids, despite showing specialized adaptation towards herbivory and potentially yielding phylogenetically relevant information, have not received as much attention. This is in part due to the state of preservation of many diadectids, as most skulls are preserved in occlusion so that the medial and occlusal views of the mandible as well as the palatal region are largely obscured. Berman *et al.* [60] stated based on the description of the lower jaw of *D. absitus* primarily on the paratype of which the jaw has been mechanically removed from the cranium. Later, Berman *et al.* [56] could describe the lower jaw of *O. pabsti* based only on the dorsoventrally crushed skull of the holotype and heavily deformed paratypes.

Using reconstructions based on new microcomputed tomography (µCT) data, we reconstruct and redescribe the mandibular anatomy of three diadectid specimens from Bromacker and a fourth previously undescribed lower jaw specimen, as well as provide a brief update on the palatal anatomy of MNG 8747. Using newly defined and previously obscured characters, we expand on the most recent character matrix on early tetrapod relationships from Clack *et al*. [37]. We recover a sister-group relationship between Diadectomorpha and Synapsida in the majority-rule consensus tree, thus providing weak support for the placement of Diadectomorpha within Amniota. Diadectomorph interrelationships are fully resolved in the majority-rule consensus tree, while all consensus trees agree on the interrelationships of derived diadectids. Based on the results of the phylogenetic analysis, we revise the taxonomy of Diadectomorpha. Notably, by corroborating on variations in cranial anatomy previously noted by Berman *et al*. [60], we recognize a third species of diadectid from the Bromacker locality. This new species further expands on the diversity of herbivorous tetrapods in this locality and thus its unique ecosystem.

The list of institutional abbreviations and their definitions used in this study is as follows:

— MNG, Museum der Natur Gotha, Gotha, Germany
— YPM, Yale Peabody Museum of Natural History, New Haven, Connecticut, USA
— UCPM, University of California Museum of Paleontology, Berkeley, California, USA
— MCZ, Museum of Comparative Zoology, Harvard, Cambridge, Massachusetts, USA
— IVPP, Institute of Vertebrate Paleontology and Paleoanthropology, Chinese Academy of Sciences, Beijing, China.

## 2. Material and Methods

### 2.1. Specimens described

Four diadectid specimens from the Bromacker locality (figure 1) were examined using µCT. This includes the holotype of *O. pabsti* MNG 10181 [56], the holotype MNG 8853 of *D. absitus* [60] and two additional specimens assigned to *D. absitus*: MNG 8747 [60] and MNG 14473 [62]. We also examined the holotype YPM 811 of the non-diadectid diadectomorph *Limnoscelis paludis*, for comparative purposes as a non-herbivorous outgroup.

### 2.2 Microcomputed tomography scanning

The holotype skull of *L. paludis* YPM 811 was scanned at the Yale Peabody Museum of Natural History in New Haven (CT, USA), by Matthew Colbert and Dave Edey using a North Star Imaging (NSI) CT Scanner with a Fein Focus High Power source. Prior to scanning, the skull was separated into five pieces along its natural fractures. Both the left lower jaw fragment and the symphysis were scanned with 170 kV, 120 µA, 2400 projections resulting in a 91.3 µm voxel size.

The remaining specimens were scanned at the Museum für Naturkunde Berlin, Germany, using an YXLON FF35 CT scanner. All scans were performed using the Cone Beam stop and go method. The skull of MNG 8853 was scanned with 220 kV, 200 µA and 1500 projections resulting in a voxel size of 88.6 µm. The skull of MNG 14473 was scanned with 180 kV, 320 µA and 4611 projections, resulting in a voxel size of 55.6 µm. The cranium and lower jaw of MNG 8747 were scanned separately. The cranium was scanned with 200 kV and 250 µA and 3222 projections, resulting in a 45.7 µm voxel size. For the lower jaw, 110 kV, 120 µA and 3226 projections were used to attain a 23.5 µm voxel size. The skull of MNG 10181 was scanned with 215 kV, 190 µA and 2398 projections, resulting in a voxel size of 50.2 µm. The SpiralFDK algorithm was used to reconstruct each specimen except for MNG 8853, which was reconstructed using Feldkamp. All scans were then segmented and visualized using the software VGStudio Max v.3.3. All raw scan data are made available on MorphoSource (MorphoSource ID: 000594326).

### 2.3. Phylogenetic analysis

We used the character–taxon matrix from Clack *et al*. [37] as a basis for the updated and expanded matrix used in this study. The subsequent compound characters from Clack *et al.* [37] were split following Brazeau [63]: 7, 22, 62, 82, 103, 111, 112, 113, 123, 135, 136, 146, 156, 160, 180, 195, 196, 210, 236, 238, 240, 284, 285. Three new characters were added or modified from Kissel [40]. Finally, we defined 22 additional characters, encompassing the observed variation in the diadectomorph mandible. Both the complete list of characters (electronic supplementary material, S1) and an overview of the new characters, operational taxonomic units (OTUs), as well as the changes we made to characters and OTUs (electronic supplementary material, S2) are included in the electronic supplementary material.

The character–taxon matrix was analysed in PAUP v.4.0a169 [64] by conducting a heuristic search (10.000 replicates using fast stepwise addition) with tree bisection and reconnection branch swapping to search for trees. Parsimony was set as the optimality criterion and all branch lengths of less than zero were set to collapse. *Whatcheeria deltae* was assigned as the outgroup. Bootstrap and Bremer decay analyses were performed to determine the support values for each node.

### 2.4. Clarification of anatomical terminology

Historically, the anatomical terminology used has varied between descriptions of diadectid jaw specimens, largely concerning the various fenestrae. In the first detailed description of a diadectid jaw, Welles [65] labelled an anterior fenestra, a small fenestra between the dentary and splenial that is recovered as unique to seymouriamorphs and diadectomorphs. Consequently, Welles [65] labelled the Meckelian fenestra as the ‘medial fenestra’ and the adductor fossa as the ‘posterior fenestra’. Some later authors adopted the terminology of medial and posterior fenestrae [60]. However, we prefer to use the more widely used terms Meckelian fenestra and adductor fossa, for comparisons with other tetrapod jaws.

## 3. Results

### 3.1. Updated mandibular anatomy of Diadectomorpha

#### 3.1.1. Limnoscelis paludis

Fracasso [66] and Berman *et al.* [45] previously described the mandible of *L. paludis* YPM 811 in detail. Due to the fragmentary nature of the specimen, the skull could be taken apart in both studies to allow the description of the jaw in medial view. Moreover, transverse breaks in the right and left mandibular rami, along the 6th and 10th tooth positions, respectively, allowed for observations of internal structures to some degree. However, the maxilla obscured the tooth row and the articular surface remains covered by the quadrate. Here, we provide additions to the description of Berman *et al.* [45], based on the reconstructions of the left jaw from µCT data.

**Figure 2.**
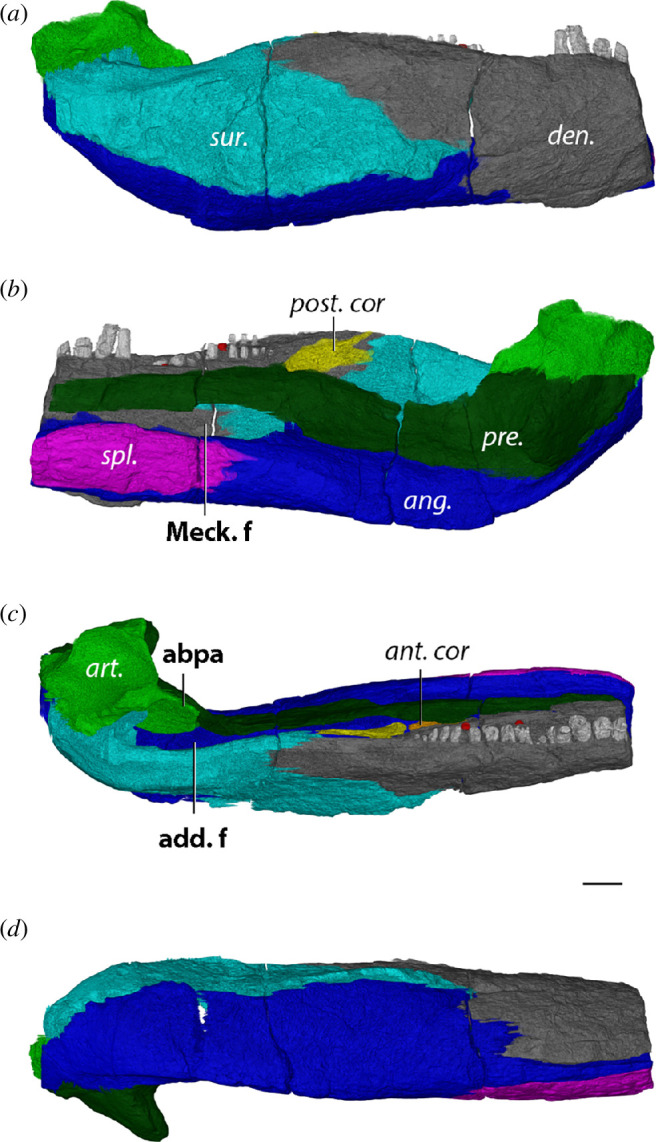
Left jaw ramus (mirrored) of *Limnoscelis paludis* YPM 811 in (*a*) lateral, (*b*) medial, (*c*) dorsal and (*d*) ventral views. Bone names in italics, anatomical structures in bold. ang., angular; ant. cor., anterior coronoid; art., articular; den., dentary; post. cor., posterior coronoid; pre., prearticular; spl., splenial; sur., surangular; abpa, anterior blade-like process of articular; add. f., adductor fossa; Meck. f., Meckelian fenestra. Scale bar, 1 cm.

**Figure 3.**
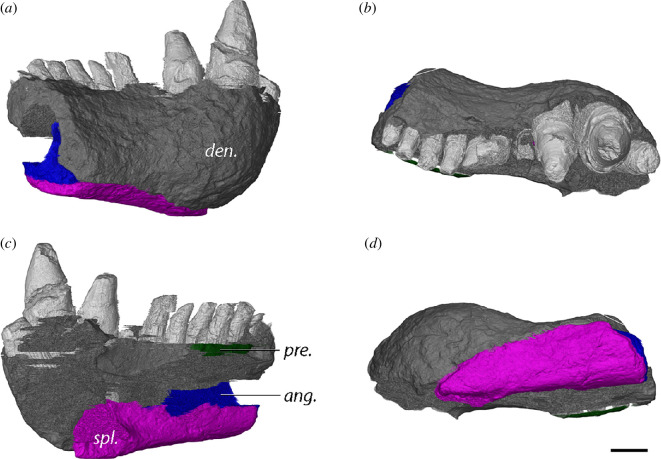
Left symphyseal region (mirrored) of *L. paludis* YPM 811 in (*a*) lateral, (*b*) dorsal, (*c*) medial and (*d*) ventral views. Bone names in italics, anatomical structures in bold. ang., angular; den., dentary; pre., prearticular. Scale bar, 1 cm.

The dentary has 25 identifiable tooth positions running in a straight line parallel to the labial margin, of which the anterior three teeth are significantly larger than the posterior teeth (figures 2 and 3). These anterior teeth are thecodont with deeply implanted roots, in particular, the roots of teeth 2 and 3 extend to the dorsal surface of the splenial and cause the lateral surface of the symphysis to appear swollen (figure 3). Beyond the first three teeth, the posterior teeth are similar in size and lack the deep roots of the anterior teeth. Three replacement teeth are present in the dentary at tooth positions 4, 16 and 20; the latter is just about to erupt. Moreover, some erosion is present at the lingual edge of the root of tooth 2, likely to host a new replacement tooth. The dentary of *L. paludis* does not bear a posterodorsal process received by the surangular, unlike what is observed in diadectids (figure 2*a*). In addition, the bone does not form an anterior foramen with the splenial at its anteroventral contact (figure 2*b*).

The anterior coronoid sits dorsally to the prearticular in the reconstruction of Berman *et al.* [45], but here we find the prearticular obscuring the anterior coronoid in the medial view (figure 2*b*). We could not observe the shagreen of denticles present on both coronoids [45] in our digital reconstruction either, which is likely due to suboptimal resolution. Berman *et al.* [45] further interpreted the surangular as its sole contributor, although close apposition of the maxilla obscures this structure. We find the dentary making up most of the apex of the coronoid eminence, with the surangular only contributing to the posterior slope (figure 2*a,b*).

The prearticular appears like a straight beam in medial view and defines the dorsal margin of an elongated but narrow Meckelian fenestra (figure 2*b*). Posteromedially, the prearticular bends around the articular and forms its ventromedial wall until its posterior extremity (figure 2*c*)—where it forms a straight transverse suture with the angular (figure 2*b*).

The glenoid surface of the articular consists of two obliquely anteroposteriorly oriented concave surfaces of roughly equal size—with the medial surface positioned slightly more anterior than the lateral surface (figure 2*c*). A ridge separating both surfaces is orientated approximately 45° with respect to the long axis of the jaw (figure 2*c*), nearly parallel to the parasagittal plane. The anterior blade-like process of the articular is short in *L. paludis*, extending from the lateral side of the prearticular to halfway of the adductor fossa (figure 2*c*). Moreover, the glenoid surface is raised slightly dorsally above the tooth row (figure 2*b*).

#### 3.1.2. Orobates pabsti

The jaw anatomy of *O. pabsti* has not been described in detail [56] due to the tight jaw occlusion in the holotype specimen MNG 10181 and the paratypes MNG 8760 and MNG 8980, the lateral compression in the latter two specimens, and the displacement in the paratype MNG 11134. Berman *et al.* [56] noted that the mandible of MNG 10181 is remarkably slender for a diadectid, summarized that the sutural pattern is akin to that of *Diadectes* and *Desmatodon*, and reported the presence of a low labial parapet. Additionally, while the articular possesses an anterior blade-like process of the articular, its extent is limited to the posterior third of the adductor fossa (labelled as ‘medial fenestra’ in [56]). We provide a more detailed description based on a digital reconstruction of the left jaw of *O. pabsti*, MNG 10181, with the aid of new µCT data.

**Figure 4.**
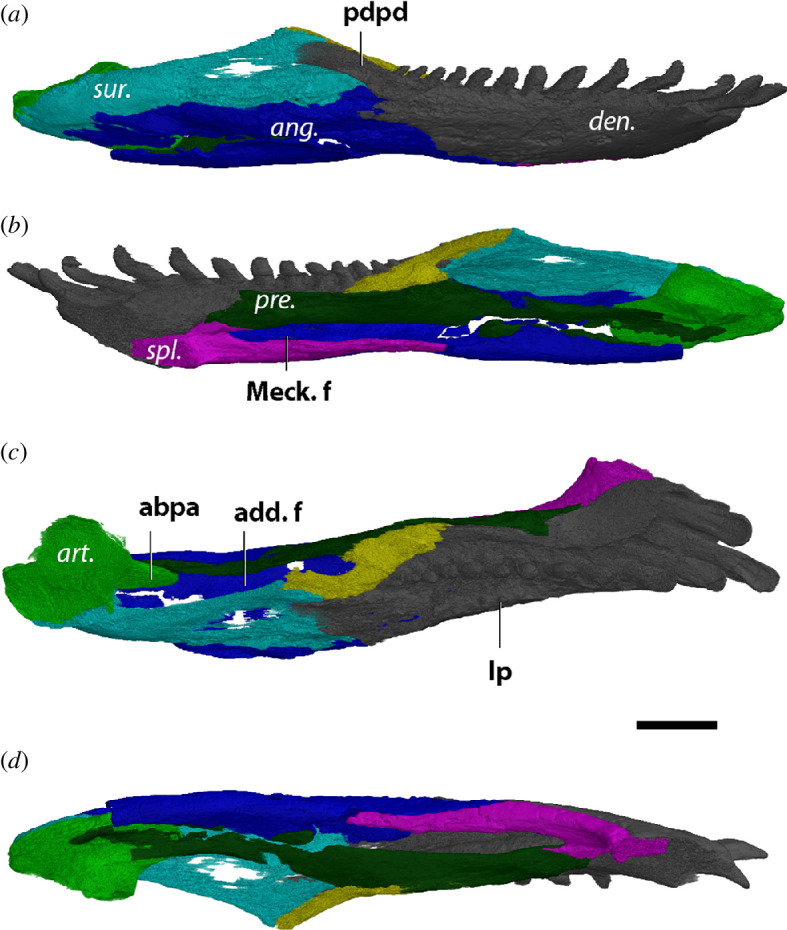
Left mandible (mirrored) of *Orobates pabsti* MNG 10181 in (*a*) lateral, (*b*) medial, (*c*) dorsal and (*d*) ventral views. Bone names in italics, anatomical structures in bold. ang., angular; art., articular; cor., coronoid; den., dentary; pre., prearticular; spl., splenial; sur., surangular; abpa, anterior blade-like process of articular; add. f., adductor fossa; lp, labial parapet; Meck. f., Meckelian fenestra; pdpd, posterodorsal process of dentary. Scale bar, 1 cm.

The left mandibular ramus is more complete than the right, as the latter has a large transverse crack running through both the surangular and angular. Despite some damage in the posterior section of the left mandibular ramus the overall structure and contacts of the individual bones can be properly described. As noted by Berman *et al.* [56], the jaw of MNG 10181 is narrow and shallow, whereby the width is consistent along its anteroposterior length (figure 4).

The dentary makes up around half the lateral surface of the mandibular ramus (figure 4*a*). It contacts the splenial ventromedially, the prearticular medially, the surangular and angular posteriorly, and the coronoid dorsomedially. Approximately two-thirds of the symphysis is made up of the dentaries (figure 4*b*), which are connected by a loose suture. The symphyseal region slopes gently and is smooth overall, apart from a few small foramina just below the tooth row on the lateral side. The dentary holds space for 13 teeth which are all erupted in MNG 10181 (figure 4). The four anteriormost teeth are spatulate, whereas the rest of the teeth are peg-like. Beyond these four spatulate anterior teeth, the tooth row appears modestly sinuous in dorsal view, meandering from the labial margin towards the coronoid lingually and then labially again along the dentary–coronoid suture (figure 4*c*). The anterior and posterior teeth are of roughly similar size, and all teeth have deeply implanted roots. Due to the poor resolution of the scan, replacement teeth could not be observed. A low labial parapet is present parallel to the posterior section of the tooth row (figure 4*c*). It presumably would have continued anteriorly but appears to be broken off. Posteriorly, near the contact with the angular, the lateral surface of the dentary becomes more rugged with anteroposterior-oriented striations. The posterodorsal process of the dentary, enveloped by the surangular, slopes upwards parallel to the coronoid (figure 4*a*).

The splenial makes up the ventral one-third of the symphysis, where it is loosely sutured to its counterpart. It is exposed mostly in medial and ventral views, forming the floor of the Meckelian fenestra (figure 4*b,d*). The splenial contacts both the dentary and the angular dorsolaterally and supports the anterior portion of the prearticular through an anterodorsal process. The contact with the dentary leaves no room for an anterior fenestra, unlike what is observed in *Diadectes sideropelicus* [65] and the other Bromacker diadectids. Apart from the presence of an anterodorsal process, the splenial is characterized as an elongated rod (figure 4*b*). The dorsoventral thickness gradually decreases towards its posterior margin at roughly the end of the tooth row.

A single coronoid sits at the dorsal apex of the jaw, forming a low, mound-like coronoid eminence along with the surangular and defines the anterior margin of the adductor fossa (figure 4*a,b*). The broad base of the coronoid rests on the prearticular ventrally, and the dorsal half meets with the dentary and surangular laterally. The anteroventral process of the coronoid is wedged between the prearticular and the dentary (figure 4*b*). There is no ossified anterior coronoid present. Due to the low resolution of the µCT scans at that region, it is not possible to discern whether a shagreen of denticles was present on the coronoid.

The angular forms a large portion of the ventral half of the mandibular ramus and is the second largest bone next to the dentary (figure 4*a*). Several cracks are present on the lateral surface. Posterolaterally, it makes up the ventral half of the jaw where it forms a suture with the surangular along its dorsal margin (figure 4*a*). The angular supports the anterior process of the articular but its full posterior extent is uncertain (figure 4*d*). Anteriorly, the angular displays a thin anteroventral process that is wedged between the dentary and splenial (figure 4*d*). The bone forms the lateral wall of both the Meckelian fenestra and the adductor fossa. Despite a large crack in the prearticular posteriorly, slivers of prearticular contact the angular posterodorsally in a straight suture (figure *8a*). The anteroventral portion of the angular meets the splenial and the dentary to form the floor of the Meckelian fenestra (figure 4*b,d*).

The surangular makes up the posterodorsal corner of the jaw and is damaged dorsally (figure 4*a*). Together with the coronoid, it comprises the coronoid eminence (figure 4*b*). Additionally, it accepts a posterodorsal process of the dentary anterodorsally (figure 4*a*). In lateral view, the surangular resembles the shape of a flattened trapezoid. The ventral margin contacts the angular, its anterior borders are defined by the dentary and the coronoid, and its posterior end wraps around the lateral surface of the articular. It contributes to the dorsal half of the lateral wall of the adductor fossa. Whether a surangular foramen is present could not be discerned.

Despite its damage posteriorly, the prearticular can still be recognized in medial view (figure 4*a*). It is an elongated rod that extends from the articular posteriorly to roughly the level of the 5th tooth position and remains mediolaterally thin throughout its length. Along this dorsal margin, the prearticular reaches the dentary shelf (figure 4*b*). A ventral process touches the dorsal surface of the splenial anteriorly. The prearticular forms the medial wall of the adductor fossa and the dorsal wall of a dorsoventrally narrow single Meckelian fenestra (figure 4*b,c*). However, this character may vary through ontogeny, as a dorsoventrally expanded Meckelian fenestra is present in the least mature paratype MNG 8980 [56]. The prearticular touches the anterior blade-like process of the articular posterolaterally, the angular posteroventrally, the base of the coronoid dorsally, the dentary anteromedially and the splenial anteroventrally. The suture with the angular is straight (figure 8*a*).

The articular is the posterior most bone of the mandibular ramus in MNG 10181. It is a small bone, mostly exposed on the dorsal and medial side (figure 4*b,c*). The glenoid surface consists of two slightly concave surfaces separated by a low median ridge. Due to damage on the medial surface, the relative anteroposterior length of the surfaces could not be established. However, the lateral surface has a wider lateral expansion. The medial margin of the medial surface curves slightly dorsally. The anterior end of the medial surface is positioned slightly more anterior than that of the lateral surface. Both glenoid surfaces are aligned with the long axis of the jaw, but oblique to the parasagittal plane, unlike *L. paludis*. A short anterior process is present, but does not reach into the adductor fossa (figures 4*d* and *9a*).

#### 3.1.3. Diadectes absitus

Berman *et al.* [60] described the lower jaw of *D. absitus* based predominantly on observations on the paratype MNG 8747, in which the right lower jaw is isolated and adequately preserved, being supplemented with observations on the occluded skull of the holotype, MNG 8853. Berman *et al.* [60] commented that, barring dimensions, the jaw of *D. absitus* does not deviate much from that of North American diadectids. However, Kissel [40] found significant differences between the jaws of the North American *Diadectes* and *D. absitus* to justify the assignment of *D. absitus* to a new genus (*Silvadectes*). As Kissel’s [40] PhD thesis was never formally published, however, the generic name *Silvadectes* remains a *nomen nudum*. Moreover, both Berman *et al.* [60] and Kissel [40] missed key features in the mandibles of MNG 8853 and MNG 8747. Our µCT data reveal additional anatomical features on the mandible of MNG 8853 that are distinct from MNG 8747. We therefore provide a detailed description of these mandibles separately. Lastly, we briefly describe the mandible of MNG 14473, recently assigned to *D. absitus* [62], which is overall very similar to MNG 8853 but shows features that were not preserved in MNG 8853.

##### 3.1.3.1. MNG 8853

**Figure 5.**
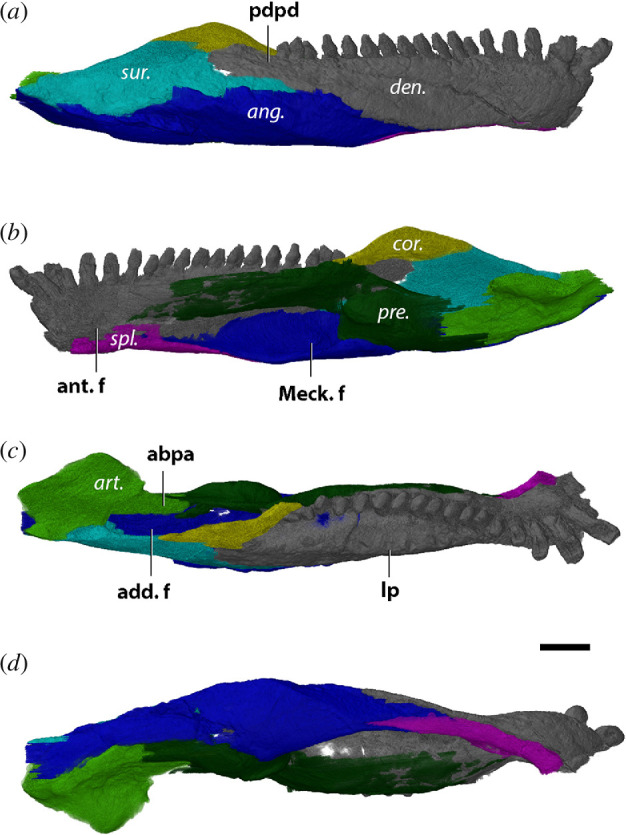
Right mandible of *Diadectes absitus* MNG 8853 in (*a*) lateral, (*b*) medial, (*c*) dorsal and (*d*) ventral views. Bone names in italics, anatomical structures in bold. ang., angular; art., articular; cor., coronoid; den., dentary; pre., prearticular; spl., splenial; sur., surangular; abpa, anterior blade-like process of articular; add. f., adductor fossa; ant. f., anterior fenestra; lp, labial parapet; Meck. f., Meckelian fenestra; pdpd, posterodorsal process of dentary. Scale bar, 1 cm.

Barring some damage on the medial surface of the jaw, the right mandibular ramus of MNG 8853 is complete. The dentaries make up most of the symphysis (figure 5*b*), where they are tightly sutured together. Apart from a few foramina just below the anterior teeth, the lateral side of the symphyseal region is smooth (figure 5*a*). The dentary bears the maximum capacity of 17 teeth, of which the anterior 4 are procumbent and incisiform, narrowly spaced and broadly concave on the distal portion of their lingual surface. Further down the tooth row, the tightly spaced cheek teeth bear a tall cusp accompanied by labial and lingual ‘shoulders’ that are oriented perpendicular to the long axis of the jaw. Six replacement teeth are present, at tooth positions 2, 4, 7, 9, 11 and 13. In the dorsal view, the tooth row resembles one period of an asymmetrical sine wave; the first half period is composed of the anterior 5 teeth whereas the second half period is stretched out and contains the rest of the cheek teeth (figure 5*c*). The amplitude of this wave is greater than in *O. pabsti*. A laterally extended dorsally flat shelf runs along the tooth row, which ends upwards in a low labial parapet. The posterodorsal process of the dentary separates the anterior part of the surangular from the coronoid (figure 5*a*). In other diadectids, this process is wedged by the surangular, so that the anterodorsal portion of the surangular supports the coronoid. This condition cannot be confirmed in MNG 8853 due to the low resolution deeper inside the skull. The dentary touches the splenial anteroventrally, the angular ventrally, the prearticular medially, the surangular posteriorly and the base of the coronoid posterodorsally.

A small splenial is exposed on the ventral side of the jaw (figure 5*b,d*). It makes up about a fifth of the symphyseal surface. The splenial is dorsoventrally thin and tapers out posteriorly until the ventral portion of the Meckelian fenestra where it is wedged by the angular. A small dorsal process nearly touches the anterior part of the prearticular, but the contact is not preserved (figure 5*b*). Nonetheless, the rounded excavation of the anterior foramen wedged between the dentary, splenial and prearticular is present (figure 5*b*).

Positioned dorsally, the coronoid is the sole contributor to a low mound-like coronoid eminence (figure *5a,b*). It is a roughly triangular-shaped bone in lateral view. The anterior process of the coronoid rests on the prearticular ventrally, at the level of the two posterior most teeth. Ventrally, the coronoid contacts the surangular and dentary.

The surangular is exposed laterally on the posterodorsal section of the mandible in MNG 8853, supporting the coronoid dorsally and the lateral facet of the articular posterodorsally (figure 5*a*). The precise contact with the coronoid could not be reconstructed due to the low contrast in that region but it is apparent that the coronoid alone makes up the coronoid eminence. A thin anterior process of the surangular wedges between the angular and the posterodorsal process of the dentary, but otherwise the surangular defines the posterodorsal margin of the dentary (figure 5*a*). Ventrally, the surangular lays on the angular with which it forms an irregular suture.

The angular contributes to a large portion of the ventral surface of the jaw of MNG 8853 (figure 5*d*). It extends from two narrow anterior processes wedged between the dentary and the splenial, at the anterior margin of the Meckelian fenestra, to the posterior margin of the articular posteriorly. Laterally, it contacts the articular, prearticular and splenial dorsally, whereas the dorsomedial contacts are the dentary and the surangular. The contact with the prearticular is formed by a straight suture (figure *8b*).

As a result of the dorsally excavated Meckelian fenestra, the prearticular of MNG 8853 appears curved (figure 5*b*). The posterior portion forms the posterior wall of the Meckelian fenestra, receiving the anterior blade-like process of the articular medially. The maximum mediolateral width of the prearticular is achieved at the anterior half of the Meckelian fenestra, forming a shelf (figure 5*c*). Dorsally, the prearticular in MNG 8853 extends to the ventral base of the tooth row (figure 5*b*). While it seems the prearticular would contact the splenial anteroventrally, this section is broken and the contact cannot be confirmed.

Posteriorly, the articular supports the jaw joint. The glenoid surfaces are anteroposteriorly elongated and roughly the same length, although the lateral surface is wider (figure 5*c*). A slight convex ridge separates the surfaces. Both surfaces are orientated parallel to the long axis of the jaw as in *O. pabsti*. The medial surface is positioned more anterior than the lateral surface, the posterior part of the medial surface ending roughly halfway the lateral surface. A prominent anterior blade-like process of the articular is projecting anteriorly to the level of the coronoid eminence but appears to be broken off anteriorly (figures 5 and *9c*).

**Figure 6.**
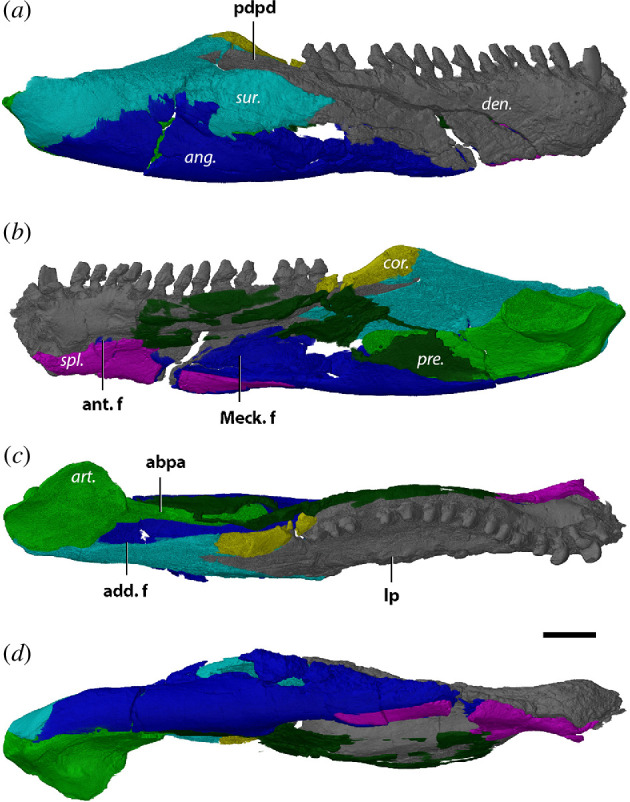
Right mandible of *Diadectes absitus* MNG 14473 in (*a*) lateral, (*b*) medial, (*c*) dorsal and (*d*) ventral views. Bone names in italics, anatomical structures in bold. ang., angular; art., articular; cor., coronoid; den., dentary; pre., prearticular; spl., splenial; sur., surangular; abpa, anterior blade-like process of articular; add. f., adductor fossa; ant. f., anterior fenestra; lp, labial parapet; Meck. f., Meckelian fenestra; pdpd, posterodorsal process of dentary. Scale bar, 1 cm.

##### 3.1.3.2. MNG 14473

Klembara *et al.* [62] described parts of the external cranial anatomy of MNG 14473 and assigned it to *D. absitus*. We agree with this assignment. Similarly, the lower jaw resembles that of MNG 8853, except for the dentary having a capacity for 16 teeth. The surangular contributes to the posterior slope of coronoid eminence (figure 6*b*). The angular does not wrap around the posterior extremity of the articular (figure 6*d*).

In addition, a thin process of the surangular wraps around the posterodorsal process of the dentary dorsally and contacts the base of the coronoid (figure 6*a*). The dorsal process of splenial contacts the prearticular anteroventrally (figure 6*b*). An anterior foramen is present between the dentary and splenial (figure 6*b*). The angular–prearticular suture is weakly interdigitated (figure 8*c*). The anterior blade-like process of the articular is completely preserved in MNG 14473, which contacts the prearticular medially and curves anterodorsally (figures 6*d* and 9*d*). The dorsoventral height is consistent throughout its length, until it rounds off at its anterior extremity.

### 3.1.4. MNG 8747

MNG 8747 was initially recovered as an occluded skull [67], but the mandible was separated from the cranium prior to formal description [60,68]. Berman *et al.* [60] noted few differences between the skull roof of MNG 8853 and MNG 8747. They interpret MNG 8747 as an immature individual of *D. absitus*, based on incomplete ossification of the distal plate of the stapes and a high degree of relief on the skull table—similar to that in juvenile North American diadectids. Moreover, the mandible of MNG 8747 has a subcircular cross-section just posterior to the symphyseal area, whereas in MNG 8853 the dorsoventral depth is greater than its mediolateral width. This, according to Berman *et al.* [60], leads to a near-dorsal projection of the adductor fossa and primary ventral projection of the Meckelian fenestra in MNG 8747. In contrast, the openings of both the adductor fossa and the Meckelian fenestra are discernible in the medial view in MNG 8853. Berman *et al.* [60] interpret the variability in the projection of these structures to reflect different ontogenetic stages. However, as the ventral margin of the mandible in MNG 8747 is broken off, the ventral extent, as well as the purported ventral projection of the Meckelian fenestra, is uncertain. We note significant variation between the mandible of MNG 8747 and the other two specimens assigned to *D. absitus*: MNG 8853 and MNG 14473. The list of the observed variations between the skulls of MNG 8853 and MNG 8747 is presented in table 1.

**Table 1.** Observed anatomical variations between MNG 8853 and MNG 8747.

trait	character	MNG 8853	MNG 8747	source
features as high degree of relief, particularly on the table portion, where well-developed, irregular prominences are created by a network of prominent, smooth channels or grooves	—	less developed	more developed	Berman *et al.* [60]
shape of projection of the posterior margin of the parietal partially separates the tabular and supratemporal	—	broad	spike-like	Berman *et al.* [60]
slope of posterior portion of the postparietal ventrally from the skull table	—	vertically	at 45°	Berman *et al.* [60]
extend of the prefrontal extends beyond the level of the anterior margin of the frontal	—	greater	lesser	Berman *et al.* [60]
ventral surface of the transverse flange of the pterygoid	—	textured	smoothly finished	Berman et al. [60]
contact between posteromedial process of the palatine and transverse flange of the pterygoid	—	achieved by a short, complementary process of pterygoid	nearly	Berman *et al.* [60]
articular surface of the occipital condyle	—	essentially flat	deep, central notochordal pit	Berman *et al.* [60]
ossification of distal plate of the stapes (‘ossified plate’)	—	completely ossified, occupying nearly the entire otic notch	partially ossified	Berman *et al.* [60]
location of dorsalmost point of maxilla in lateral aspect	31	approximately at its mid-length (1)	in anterior third of maxilla length (0)	Clack *et al.* [37]/this study
parietal–postparietal sutural course	41	smooth (0)	interdigitating (1)	Clack *et al.* [37]/this study
if a single, large elongated Meckelian fenestra is present	215	dorsoventrally expanded, excavating the prearticular (1)	dorsoventrally narrow (0)	this study
contribution of the supratemporal and tabular to the skull table	304	supratemporal forms most of the skull table (1)	both occupy subequal portions (2)	Berman *et al.* [60] and Clack *et al.* [37]
labial parapet	306	present but low (1)	absent (0)	Berman *et al.* [60], Clack *et al.* [37]/this study
if a coronoid eminence is present, its dorsal surface is	324	rounded (0)	dorsally pointed (1)	this study
presence of small anteriorly oriented foramen or foramina on posterolateral surface	325	absent (0)	present (1)	this study
prearticular, dorsal extent	326	level with base of tooth row (1)	ventral to base of tooth row (0)	this study
angular–prearticular sutural course	334	smooth	interdigitated	this study
palatal replacement teeth	—	present	absent	this study

**Figure 7.**
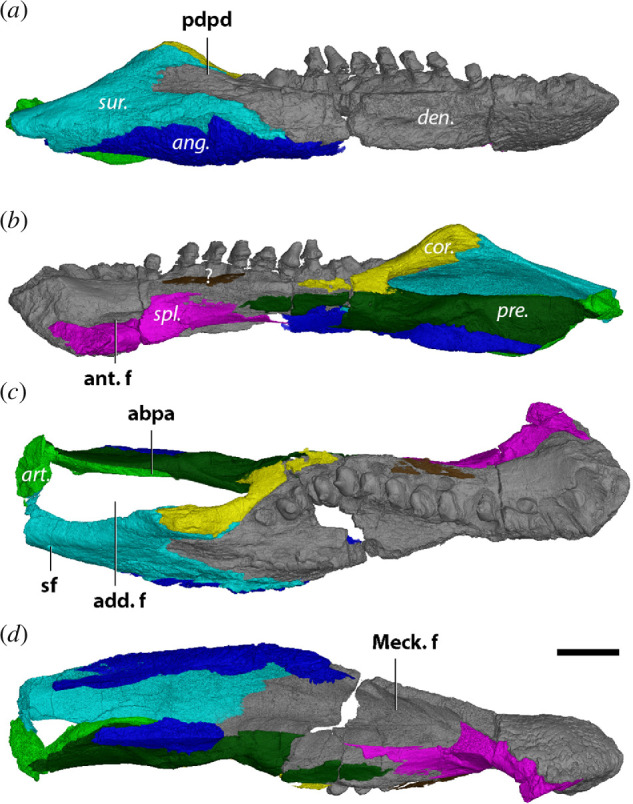
Right mandible of *Diadectes dreigleichenensis* MNG 8747 in (*a*) lateral, (*b*) medial, (*c*) dorsal and (*d*) ventral views. Bone names in italics, anatomical structures in bold. ang., angular; art., articular; cor., coronoid; den., dentary; pre., prearticular; spl., splenial; sur., surangular; ?, unknown bone fragment; abpa, anterior blade-like process of articular; add. f., adductor fossa; ant. f., anterior fenestra; pdpd, posterodorsal process of dentary; sf, surangular foramen. Scale bar, 1 cm.

The dentary makes up more than half of the lateral surface of the mandibular ramus (figure 7*a*). Anteriorly, the symphyseal area is rugose, and several foramina are noticeable just below where the incisiform teeth would be. There is a capacity for a row of 16 dentary teeth, with replacement teeth present at teeth 2, 4, 7, 9, 10, 12 and 15. All sockets are occupied, but the crowns of the four anterior teeth as well as the posterior most tooth are broken off. The tooth row of MNG 8747 forms a single sine wave in dorsal view, curving from the labial anterior teeth to the coronoid lingually and labially again following the dentary–coronoid suture (figure 7*c*). The cheek teeth are transversely expanded, yet the labial and lingual cusps are poorly developed, more resembling ‘shoulders’. There is no labial parapet, unlike what is present in *D. sideropelicus* [65] (therein *D. lentus*) and MNG 8853 [56,60]. Instead, a dorsally flat shelf expands mediolaterally, encompassing the tooth row (figure 7*c*). As the dentary shelf in MNG 8747 is more mediolaterally expanded than in the other diadectids, the amplitude of the sinuous tooth row in MNG 8747 is more apparent. The posterodorsal process of the dentary is received by the surangular until the level of the coronoid eminence (figure 7*a*). Ventromedially, the dentary contacts the splenial and we confirm the presence of an anterior fenestra between the dentary and the splenial (figure 7*b*). The posteromedial surface of the dentary touches the ventral base of the coronoid and the anterior portion of the prearticular. The dentary forms a suture with the surangular posteriorly that can be followed along the lateral surface of the jaw (figure 7*a*). Contact with the angular is minimal due to the ventral surface of the jaw missing (figure 7*d*).

The apex of the jaw is formed by a triangular coronoid, which is flanked by the surangular (figure 7*a*,*b*). While the surangular largely obscures the coronoid in lateral view, the coronoid does remain visible along the anterodorsal margin of the surangular. The coronoid does not bear a shagreen of denticles, in contrast to earlier branching diadectomorphs, such as *L. paludis* [45]. An unidentified thin splint of bone is attached to the medial side of the tooth row, which could possibly represent an anterior coronoid (figure 7*b*).

**Figure 8.**
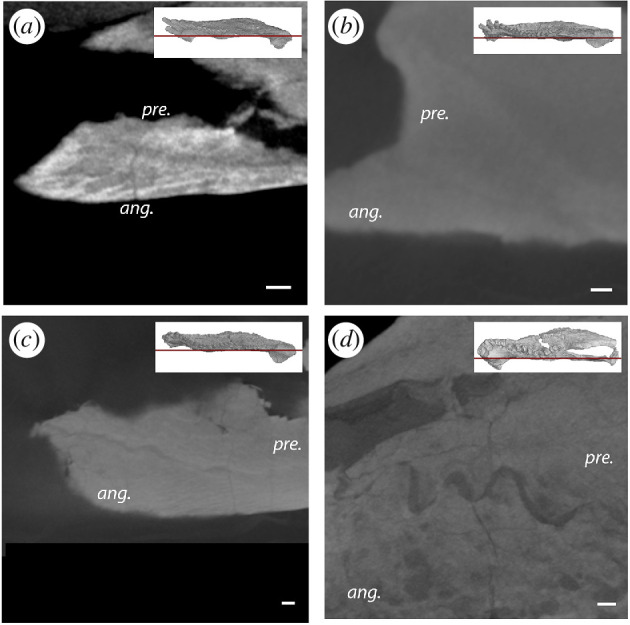
Virtual thin sections showing the angular–prearticular suture morphology in the mandibles of (*a*) *Orobates pabsti* MNG 10181, (*b*) *D. absitus* MNG 8853, (*c*) *Diadectes absitus* MNG 14473 and (*d*) *Diadectes dreigleichenensis* MNG 8747. Note the straight suture in (*a*,*b*), the weakly interdigitated suture in (*c*) and the strongly interdigitated suture in (*d*). ang., angular; pre., prearticular. Scale bar, 1 mm.

A small ventromedial fragment of bone was identified by Berman *et al*. [60] (fig. 10*d* therein) as part of the surangular. Given the ventral position of this fragment, we instead identify it as part of the angular (figure 7*b*). This piece sutures with the prearticular dorsally and forms a ventromedial contact with the anterior blade-like process of the articular. At the level of the coronoid eminence, the angular–prearticular suture is strongly interdigitated (figures 7*b* and 8*d*), unlike the straight angular–prearticular suture in the other diadectomorphs. The lateral angular fragment supports the ventral margin of the surangular and the posteroventral part of the dentary.

Both the coronoid and surangular contribute to the coronoid eminence (figure 7*b*,*c*). The surangular is exposed on the posterolateral side of the jaw, where it composes about the dorsal two-thirds of the lateral surface. In lateral view, it is a flattened roughly triangular bone. A dorsoventrally thin process is wedged in between the angular and the posterodorsal process of the dentary (figure 7*a,c*). The surangular supports the dorsal most portion of the coronoid that forms the coronoid eminence dorsally, contacts the dentary anteriorly and the angular ventrally. A thin posteromedial fragment is close to touching the base of the glenoid surface of the articular (figure 7*c*), which presumably would be the case if the articular was more complete. A single anterodorsally oriented surangular foramen is positioned on the posterodorsal side of the surangular (figure 7*c*). Based on the location and proximity to the articular, it is tentatively interpreted as the foramen auriculotemporalis.

**Figure 9.**
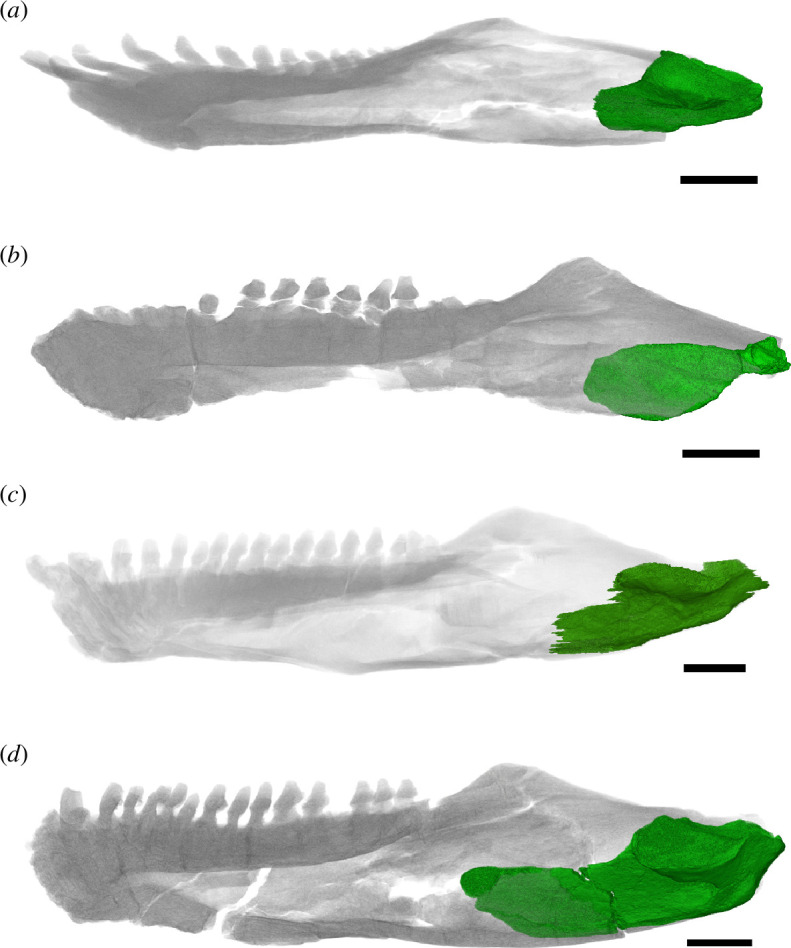
Extend of anterior blade-like process of articular (in green) in various diadectids, shown with the mandible transparent. (*a*) *Orobates pabsti* MNG 10181, (*b*) *Diadectes dreigleichenensis* MNG 8747, (*c*) *Diadectes absitus* MNG 8853 and (*d*) *Diadectes absitus* MNG 14473. Scale bars, 1 cm.

Medially, the prearticular is partially preserved as a straight, rod-like bone like in *O. pabsti* (figure 7*b*). This is unlike the condition observed in MNG 8853 and MNG 14473, where the dorsal excavation of the Meckelian fenestra gives the prearticular a curved appearance. The posterior section of the prearticular in MNG 8747 forms the lateral wall of the adductor fossa, while the anterior portion would define the posterodorsal roof of a narrow Meckelian fenestra. The lack of dorsal excavation of the Meckelian fenestra in contrast to MNG 8853 is striking. In *O. pabsti*, the Meckelian fenestra is dorsally excavated in the immature specimen MNG 8980, while it is narrow in the more mature MNG 10181—indicating the fenestra closes through ontogeny by increased ossification. This pattern is seemingly reversed between MNG 8853 and MNG 8747, whereby dorsal excavation is absent in the subadult MNG 8747 and is present only in the adult MNG 8853. It is unlikely that the Meckelian fenestra increases excavation through ontogeny, and so we reject that this variation is caused merely by ontogeny. The prearticular in MNG 8747 is mediolaterally expanded where it forms the medial wall of the anterior half of the adductor fossa. It contacts the angular posteroventrally, the anterior blade-like process of the articular posteromedially, the base of the coronoid anterodorsally and the dentary laterally. The suture with the angular is strongly interdigitated (figure 9*d*). Dorsally, the bone does not extend further than the contact with the base of the coronoid, ventral to the tooth row (figure 7*b*). Due to the anterior portion of the bone being missing, contact with the splenial could not be confirmed.

The articular is the posterior most bone in the jaw; however, this side is heavily damaged so that the glenoid surfaces are not preserved. A major anterior blade-like process of the articular is present, extending to about the level of the coronoid eminence (figures 7*c* and 9*b*). This blade-like process, supporting the medial side of the prearticular, is transversely thin. Its ventral margin is hemispherical, so that its maximum dorsoventral height is achieved halfway through its length. By contrast, the dorsoventral height of this process in MNG 14473 is more consistent throughout its length, until its anterior end, where its height increases. Lastly, the glenoid surfaces of the articular would have been on the same level as the tooth row, as opposed to *D. sideropelicus* in which the jaw joint is depressed with respect to the tooth row [65].

In addition to the observed differences in mandibular anatomy, Berman *et al.* [60] described the following dissimilarities in cranial anatomy between MNG 8853 and MNG 8747: the projection of the posterior margin of the parietal that partially separates the tabular from the supratemporal is broad in MNG 8853 and spike-like in MNG 8747. In MNG 8853 the supratemporal makes up the majority of the skull table, whereas in MNG 8747 it contributes roughly the same amount as the tabular. While the posterior portion of the postparietal slopes at an angle of 45° in MNG 8747, this is vertical in MNG 8853. The prefrontal extends further beyond the level of the frontal in MNG 8853 than in MNG 8747. A deep notochordal pit forms on the articular surface of the occipital condyle in MNG 8747, though this surface is essentially flat in MNG 8853. On the palatal surface, contact between the posteromedial process of the palatine and the transverse flange of the pterygoid is accomplished through a short accessory process on the pterygoid in MNG 8853, while this contact does not form in MNG 8747. Lastly, the ventral surface of the transverse flange of the pterygoid in MNG 8853 is textured and smoothly finished in MNG 8747.

On top of that, we scored MNG 8853 and MNG 8747 separately in our character matrix. In previous phylogenetic studies, both MNG 8853 and MNG 8747 were scored under the same OTU: *D. absitus* [37,39]. This meant in practice that characters that could be scored on MNG 8853 were taken from that specimen only, supplemented with scores from MNG 8747 from areas that were obscured in MNG 8853—such as the mandible and palate. As we treated MNG 8853 and MNG 8747 as separate OTUs, the following characters are positively scored differently (i.e. the character was applicable to both specimens and scored differently). The dorsal most point of the maxilla in the lateral aspect is located in the anterior third of MNG 8747 (Ch. 31, state 0), while it is located roughly at the midpoint in MNG 8853 (Ch. 31, state 1). The parietal–postparietal sutural course is smooth in MNG 8853 (Ch. 41, state 0) but interdigitated in MNG 8747 (Ch. 41, state 1). The elongated Meckelian fenestra does not excavate the prearticular in MNG 8747 (Ch. 215, state 0), but does so in MNG 8853 (Ch. 215, state 1). The labial parapet is scored as present in MNG 8853 (Ch. 306, state 1) and absent in MNG 8747 (Ch. 306, state 0). The coronoid eminence is triangular in MNG 8747 (Ch. 324, state 1) yet mound-like in MNG 8853 (Ch. 324, state 0). MNG 8747 bears a surangular foramen (Ch. 325, state 1) which is absent in MNG 8853 (Ch. 325, state 0). The prearticular extends dorsally to the level of the tooth row in MNG 8853 (Ch. 326, state 1) but not in MNG 8747 (Ch. 326, state 0). In MNG 8747, the angular forms a strongly interdigitated suture with the prearticular (Ch. 334, state 2), whereas this suture is straight in MNG 8853 (Ch. 334, state 0). Berman *et al.* [60] observed that the anterior extend of the prefrontal relative to the frontal is much more apparent in MNG 8853 (Ch. 336, state 1) than in MNG 8747 (Ch. 336, state 0). Lastly, as noted by Berman *et al.* [60], the parietal bears a posteriorly oriented spike-like process in MNG 8747 (Ch. 341, state 1), which is absent in MNG 8853 (Ch. 341, state 0). The states of characters 31, 41, 215, 306, 324, 325, 334 and 341 are furthermore recovered as unambiguous apomorphies in the majority-rule consensus tree (table 2).

**Table 2.** Unambiguous apomorphies of selected taxa in the majority-rule consensus tree.

taxon	apomorphies (unambiguous)	total
*D. dreigleichenensis*	31(0), 41(1), 215(0), 306(0), 324(1), 325(1), 334(2), 341(1)	8
*D. dreigleichenensis + D. absitus*	153(1)	1
*Diadectes*	3(1), 144(0)	2
*Kuwavaatakdectes sanmiguelensis*	18(1), 37(1), 43(0), 52(1), 90(1), 253(1)	6
Diadectidae	184(1), 221(1), 308(1)	3
Diadectomorpha	10(1), 71(0), 97(0), 214(1), 233(1), 247(0),258(1), 283(0), 311(1), 331(0), 336(0)	9
Diadectomorpha + Synapsida	5(1), 29(0), 42(1), 92(1), 95(1), 148(1), 222(2), 223(2), 248(0), 260(0), 265(1), 318(4), 332(1)	13
*Alveusdectes* + Eothyrididae	52(1), 68(0), 98(1), 101(2), 112(0), 321(1), 341(1)	7
*Dimetrodon + Varanops*	58(1), 91(1), 192(1), 218(1), 230(0), 304(2)	6
Synapsida	45(1), 122(1), 164(1)	3
Sauropsida	39(1), 62(1), 98(1), 109(1), 169(1)	5
Amniota	47(1), 103(2), 219(1), 304(1), 312(1)	5

Berman *et al.* [60] interpreted MNG 8747 as a subadult individual and MNG 8853 as an adult individual. It is not likely that ontogeny alone explains all observed variation between MNG 8747 and MNG 8853. For instance, two characters describe the complexity of the parietal–postparietal and the angular–prearticular suture (characters 41 and 334, respectively). For both characters, the condition in MNG 8747 is more complex than in MNG 8853, while the sutural course only gains more complexity through ontogeny [69]. Additionally, it is not likely the surangular foramen closes through ontogeny. A similar character has been used in phylogenetic studies [70], and its presence is noted in various adult sauropsids [70–72]. There is little documentation on this structure in synapsids, unfortunately.

Additionally, as noted, MNG 8747 has faced dorsoventral crushing. It is not likely that this crushing has caused the discrepancy in Meckelian fenestra morphology. The character describes the dorsal excavation of the Meckelian fenestra into the prearticular. When the Meckelian fenestra is dorsally excavated, it is expected to see the portion of the prearticular anterior to the angular–prearticular suture sloping dorsally strongly as in MNG 8853 and MNG 14473 (figures 5*b* and 6*b*). In MNG 8747, this section of the prearticular is horizontal and does not display any fractures (figure 7*b*).

### 3.2. Remarks on the palatal anatomy in Diadectidae

#### 3.2.1. Updated description of MNG 8747

**Figure 10.**
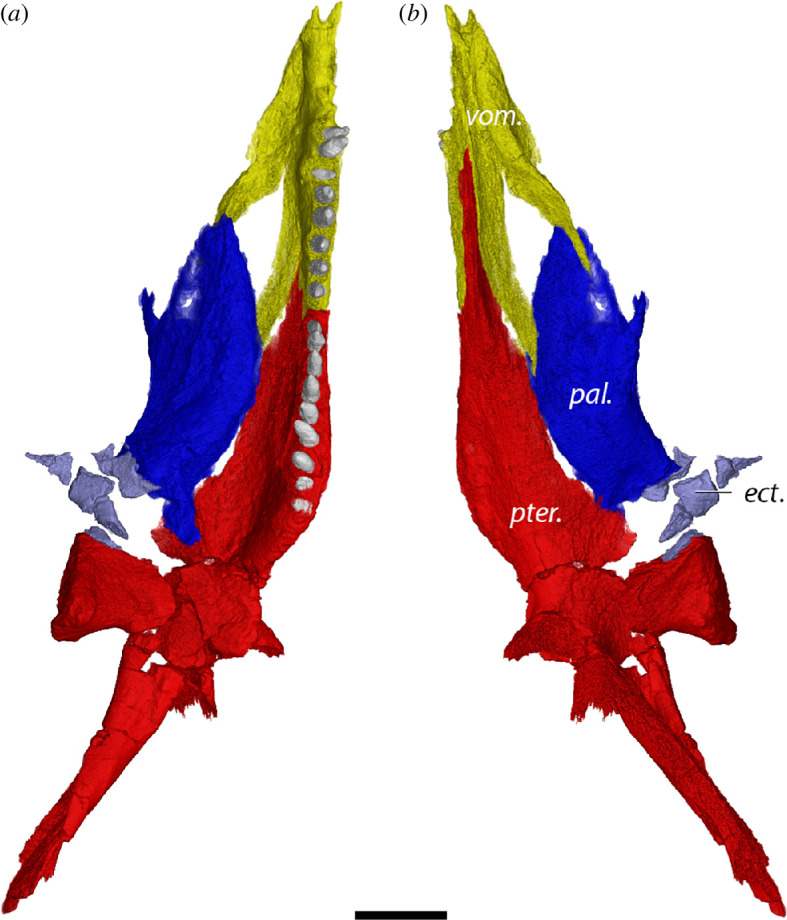
Palatal anatomy of *D. dreigleichenensis* MNG 8747 in (*a*) ventral and (*b*) dorsal views. ect., ectopterygoid; pal., palatine; pter., pterygoid; vom., vomer. Scale bar, 1 cm.

As the cranium has been freed from the mandibles in MNG 8747 [60], the palatal region is clearly visible in the ventral view. Since this region is poorly visible in occluded skulls, it is poorly known [73] and the description of Berman *et al.* [60] contributed greatly to the knowledge of the palatal area in Diadectidae. The reconstruction of Klembara *et al.* [62] follows the description of Berman *et al.* [60]. A single highly organized row of conical teeth is present in the palate (figure 10*a*), as in all diadectomorphs except for *L. paludis* [74]. Outside of the Diadectidae + *Tseajaia* clade, this arrangement of pterygoid dentition is known only from squamates like iguanids, snakes and mosasaurs—although in these groups, the pterygoid teeth are more commonly recurved [75–77].

Our segmentation largely follows previous reconstructions. However, in contrast to Berman *et al.* [60] and Klembara *et al*. [62], we find that the vomer contacts the palatine (figure 10). The vomer bears eight conical teeth, the pterygoid bears further nine teeth.

#### 3.2.2. Palatal tooth replacement pattern

**Figure 11.**
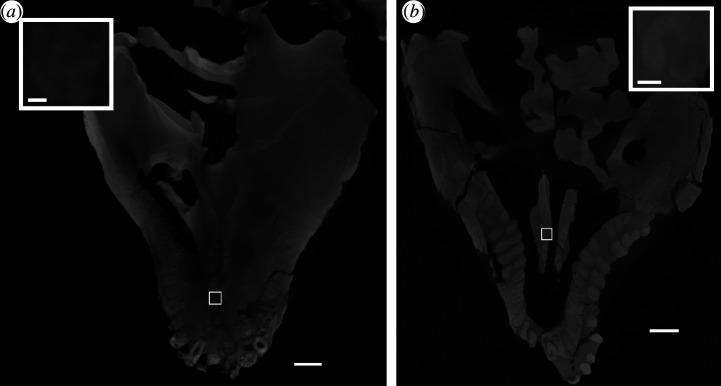
Palatal replacement teeth in (*a*) *Diadectes absitus* MNG 8853 and (*b*) *Diadectes absitus* MNG 14473. Scale bar, 1 cm.

More notably, we also discovered minute replacement teeth alongside the palatal dentition (figure 11). MNG 8853 bears a replacement tooth at the anteriormost palatal tooth, positioned slightly distolingual to the active tooth (figure 11*a*). In MNG 14473, a replacement tooth is present mesiolingual to the 9th active tooth (figure 11*b*). In both specimens, these replacement teeth are found only in the right palatal ramus. The mode of palatal tooth replacement in these diadectids appears to resemble that of diadectid cheek teeth replacement described by LeBlanc & Reisz [78], albeit at a much slower pace. No palatal replacement teeth were observed either in *O. pabsti* MNG 10181 or in *D. dreigleichenensis* MNG 8747. Given the apparent slow rate of replacement, it cannot be stated whether the absence of palatal replacement teeth in these specimens reflects an actual absence of palatal tooth replacement in their respective taxon or merely the absence of palatal replacement teeth at the time of these individuals’ death. Nonetheless, this pattern of replacement differs greatly from the typical replacement pattern of palatal dentition in other Palaeozoic tetrapods, which is characterized by the overgrowth of new layers of bone to which the new functional denticles attach [79].

### 3.3. Phylogenetic analysis

**Figure 12.**
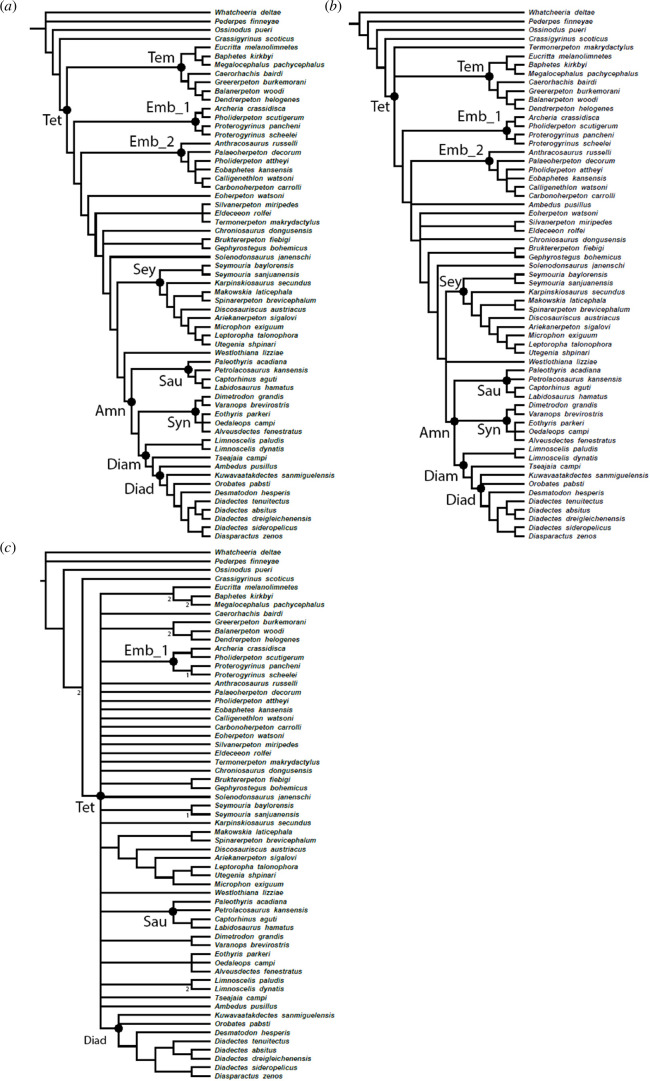
Cladogram showing consensus trees of 702 most parsimonious trees. (*a*) Majority-rule consensus tree, (*b*) Adams consensus tree and (*c*) strict consensus tree. Bootstrap values over 50 are reported in the majority-rule tree, Bremer decay values are reported in the strict consensus tree. Tet., Tetrapoda; Tem., Temnospondyli; Emb_1,. Embolomere clade 1; Emb_2, Embolomere clade 2; Sey., Seymouriamorpha; Amn., Amniota; Syn., Synapsida; Sau., Sauropsida; Diam., Diadectomorpha; Diad., Diadectidae.

The phylogenetic analysis produced 702 optimal trees, each with a total branch length of 1499 (figure 12). The majority-rule consensus tree recovers a fully resolved Diadectomorpha within Amniota. Specifically, diadectomorphs are found as the sister-group to Synapsida. Sauropsida (*Paleothyris acadiana* + (*Petrolacosaurus kansensis* + (*Captorhinus aguti + Labidosaurus hamatus*))) forms the sister-group to Diadectomorpha + Synapsida. The sister-group to Amniota is formed by *W. lizziae* followed by Seymouriamorpha. The topology of Amniota is supported by five unambiguous apomorphies, while a Diadectomorpha + Synapsida sister-relation is supported by 13 unambiguous apomorphies (table 2). In both the majority-rule consensus and the Adams consensus, Diadectomorpha is a monophyletic clade with Limnoscelidae (*L. paludis + L. dynatis*) as its earliest branching clade (figure 12*a,b*). This monophyletic Diadectomorpha is further supported by nine unambiguous apomorphies.

The majority-rule consensus tree further resolves Diadectomorpha (figure 12*a*). Limnoscelidae and Tseajaiidae are the outgroups to Diadectidae. The purported diadectid *Ambedus pusillus* [80] is recovered as the earliest branching diadectid, followed by *‘Diadectes’ sanmiguelensis*, *O. pabsti* and *D. hesperis*. Then, there is a split between two clades of derived diadectids. MNG 8853 and MNG 8747 are recovered as sister taxa, with *Diadectes tenuitectus* as their outgroup. The other derived diadectid clade is formed by *D. sideropelicus* and *Diasparactus zenos. Alveusdectes fenestratus*, another purported diadectid [81], is recovered within synapsids instead, in a polytomy with the eothyridids *Eothyris* and *Oedaleops*.

The Adams consensus (figure 12*b*) is overall very similar to the majority-rule consensus tree, but less well resolved regarding the interrelationships of Diadectomorpha, Amniota and their direct sister-groups. Derived diadectids form a polytomy with *O. pabsti* and *‘D.’ sanmiguelensis*. Moreover, diadectomorphs fall in a polytomy with Sauropsida and Synapsida. The direct sister-group to this clade is unclear, as it forms a polytomy with Seymouriamorpha and *W. lizziae*. Notably, *A. pusillus* is placed far outside of Diadectomorpha in the Adams consensus, in a polytomy with a clade of Embolomeri and all taxa closer to Amniota. Lastly, *Termonerpeton makrydactylus* [37] is removed from a polytomy with *Silvanerpeton miripedes* and *Eldeceeon rolfei* into a far more basal position; at the base of Tetrapoda in a polytomy with Temnospondyli and total-group Amniota.

The strict consensus, however, collapses of all tetrapods into one large polytomy with few exceptions (figure 12*c*). Most notably, the diadectids are recovered as a clade. The interrelationships of diadectids more derived than *O. pabsti* and ‘*D’. sanmiguelensis* remain fully resolved. Additionally, we find other clades supported, e.g. Sauropsida, the eothyridid synapsids including *A. fenestratus*, Embolomeri clade 1, a subclade of Seymouriamorpha and two subclades of Temnospondyli. The two temnospondyl subclades are well supported with a Bremer support value of 2 (figure 12*c*).

Additionally, we do report a couple of notable differences between previously published topologies derived from this character matrix [37,39,61] and our majority-rule and Adams consensus trees, outside of Amniota and Diadectomorpha. Here they are listed in order of their distance to Amniota.

We recover *Greererpeton burkemorani* in a more derived position, in both the majority-rule and Adams consensus (figure 12*a,b*) [39]. Clack *et al.* [37] find *G. burkemorani* among stem-tetrapods either as sister-taxon to *Crassigyrinus scoticus* or more crown-ward than *C. scoticus* as the sister-taxon to Tetrapoda. We find *G. burkemorani* as the sister-taxon to the temnospondyls *Dendrerpeton helogenes* and *Balanerpeton woodi*, which in turn forms a sister-relation to *Caerorhachis bairdi*.

Embolomeri are recovered as paraphyletic, divided into two major clades, in both the majority-rule and Adams consensus (figure 12*a,b*). The first clade consists of (*Proterogyrinus scheelei + P. pancheni*), (*Pholiderpeton scutigerum + Archeria crassidisca*), the second of *Anthracosaurus russelli, Palaeoherpeton decorum* and a polytomy of *Pholiderpeton attheyi, Eobaphetes kansensis* and (*Calligenethlon watsoni + Carbonoherpeton carrolli*). While the contents of each subclade match with previous topologies, embolomeres are monophyletic in the studies by Klembara *et al.* [39] and Clack *et al.* [37].

The recently described *T. makrydactylus* was found as the sister-taxon to *E. rolfei* and *S. miripedes* in the implied weighting analysis of Clack *et al.* [37]. We do find an affinity of *T. makrydactylus* with *E. rolfei* and *S. miripedes*, but in our majority-rule analysis, this relationship collapsed into a polytomy (figure 12*a*). In the Adams consensus, *T. makrydactylus* is pulled out of this clade entirely into a polytomy with Temnospondyli and total-group Amniota.

Seymouriamorphs are recovered as the sister-taxon to a clade consisting of Amniota + *W. lizziae* in the majority-rule consensus (figure 12*a*). This differs from the reweighted trees of Klembara *et al.* [39] and Clack *et al.* [37], as in these trees *Solenodonsaurus janenschi* is also closer to crown Amniota than Seymouriamorpha. In addition, our topology of Seymouriamorpha differs from that of previous iterations of this character matrix [37,39,61]. Both our majority-rule consensus tree and Adams consensus place the *Seymouria* species as the earliest branching seymouriamorphs, followed by *Karpinskiosaurus secundus*, while *Utegenia shpinari* is deeply nested (figure 12*a,b*). By contrast, *U. shpinari* is consistently found as the earliest branching seymouriamorph in the other studies [37,39,61].

### 3.4. A revised taxonomy of Diadectomorpha

We consider all of the anatomical variations described above and conclude that MNG 8747 is sufficiently distinct from MNG 8853 to justify the erection of a new species (see below). The genus ‘*Diadectes*’ is recovered as polyphyletic in all consensus trees (figure 12). This is largely solved by erecting a new genus for ‘*D.*’ *sanmiguelensis* (see below). However, *Diadectes* is now rendered paraphyletic as it includes *D. zenos* as an in-group. The postcranial anatomy of *D. zenos*, including the exceptionally tall neural arches [82] is too distinct to simply synonymize *Diasparactus* with *Diadectes*. It is recommended to further investigate the

type material of *D. zenos* before additional taxonomic revisions are done, but that is outside the scope of this current study.

### 3.5. Systematic palaeontology

Order Diadectomorpha Watson 1917 [83]
Family Diadectidae Cope 1880 [84]
Genus *Diadectes* Cope 1878 [85]
*Diadectes dreigleichenensis* sp. nov.
zoobank.org:act:78B0B15B-866B-49CF-B0E6-03C7227F9626

*Holotype*: MNG 8747, greater part of cranium with isolated lower jaws [60].

*Location and stratigraphy*: MNG 8747 was excavated in 1991 at the Bromacker locality near Tambach-Dietharz in Thüringen, central Germany (figure 1), in the uppermost level of the Tambach Sandstone of the lower Permian Tambach Formation [60,67]. Precise dating of the Tambach Formation is challenging due to the lack of volcaniclastic rocks [51]. Schneider *et al.* [86] tentatively assign an Artinskian age for the Upper Rotliegend I, including the Tambach Formation based on conchostracan biostratigraphy, but it could possibly be as old as early Sakmarian/late Asselian [51,87,88].

*Diagnosis*: *Diadectes dreigleichenensis* is a medium-sized diadectid from the early Permian Bromacker locality. *Diadectes dreigleichenensis* is distinguished from its sister-taxon *D. absitus*, from the same locality, by the following apomorphic features: (i) dentary shelf lacking a labial parapet; (ii) strongly interdigitating suture between the angular and prearticular; (iii) dorsal surface of coronoid triangular; (iv) presence of surangular foramen; (v) shallow Meckelian fenestra that does not excavate into the prearticular; (vi) anterior portion of prefrontal does not extend significantly anteriorly beyond frontal; (vii) presence of a posterior spike-like process of the parietal; (viii) dorsalmost point of maxilla anterior to midline of maxilla; (ix) parietal–postparietal suture interdigitated; and (x) subequal contribution of supratemporal and tabular to skull table.

The combination of these features is unique for *D. dreigleichenensis*. A surangular foramen like in *D. dreigleichenensis* has not been observed in any diadectomorph. The lack of the labial parapet is further unique within diadectids. Whereas an interdigitated angular–prearticular suture is present in MNG 14473, *D. sideropelicus* and *D. hesperis*, these are not as strongly interdigitated as in *D. dreigleichenensis*. A triangular coronoid is shared with only *D. hesperis*. A shallow Meckelian fenestra is present in *L. paludis* and the holotype of *O. pabsti*. A spike-like posterior process of the parietal is present only in *L. paludis* and the holotype of *O. pabsti*. The subequal contribution of the supratemporal and tabular is shared with *O. pabsti* and *D. sideropelicus*. An interdigitated parietal–postparietal sutural course is otherwise only known in the two *Limnoscelis* species. The anterior-positioned dorsal apex of the maxilla is shared with *T. campi* and *A. pusillus*. Lastly, the maximum anterior extent of the prefrontal relative to the frontal is highly diverse, but the condition in MNG 8747 is also seen in *‘D.’ sanmiguelensis*, *L. paludis*, *T. campi* and the holotype of *O. pabsti*.

Additionally, *D. dreigleichenensis* shares a couple of derived diadectid features with *O. pabsti* and other members of *Diadectes* that separate *D. dreigleichenensis* from earlier branching diadectomorphs: a posterior process of the dentary, a single organized row of conical teeth on the pterygoid palatal ramus, and a dentary tooth row that appears sinusoid in occlusal view. *Diadectes dreigleichenensis* further differs from *O. pabsti* by the presence of an anterior blade-like process of the articular that extends anteriorly into the adductor fossa. On the other hand, the cheek teeth of *D. dreigleichenensis* are not as widely expanded as in taxa like *D. hesperis* and *D. sideropelicus*.

*Remarks*: Berman *et al.* [60] assigned the Bromacker diadectid specimens to the genus *Diadectes*, without justification beyond the statement that ‘there can be no doubt of the generic assignment of the Bromacker diadectid to *Diadectes*’ (p. 86). We recover two apomorphies for the genus *Diadectes*; namely, the presence of an alary process of the premaxilla and the absence of dentition on the transverse flange of the pterygoid (table 2). While the presence of the alary process cannot be confirmed in *D. dreigleichenensis* due to the damage in this region of the only known specimen MNG 8747, dentition is absent on the transverse flange of the pterygoid in MNG 8747. Additionally, the position of *D. dreigleichenensis* as sister to *D. absitus* supports its position within *Diadectes*. Few other characters are listed that differ between MNG 8853 and MNG 8747 (table 1), but these pertain to sculpturing and level of ossification and are thus more likely to reflect the ontogenetic stage.

*Etymology*: The specific epithet is after *Drei Gleichen* (German meaning: three of the same), in reference to the seemingly similar looking now three diadectids from the Bromacker locality. *Drei Gleichen* itself refers to three iconic castles from the Middle Ages, each situated on a hill top between Gotha and Erfurt within the *UNESCO Global Geopark Thüringen Inselsberg—Drei Gleichen*, which also contains the Bromacker locality. Moreover, *Drei Gleichen* is also the name of the municipality, where the authors resided during recent excavations at the Bromacker locality, as several other researchers have done since the 1990s.

Genus *Kuwavaatakdectes* gen. nov.
*Kuwavaatakdectes sanmiguelensis* comb. nov.
*Synonyms*: *Diadectes sanmiguelensis* [89], *Oradectes sanmiguelensis* [40].

*Holotype*: MCZ 2989. A partial skeleton consisting of a slightly distorted skull with several articulated cervical vertebrae, a few incomplete ribs, a partial pectoral girdle consisting of left and right clavicles, the interclavicle, left cleithrum, left scapulocoracoid and the left forelimb [40,89].

*Location and stratigraphy*: Cutler Formation of Placerville Area, San Miguel County, Colorado [89].

*Diagnosis: Kuwavaatakdectes* is unique among Diadectomorpha in the following characteristics: (i) the Meckelian fenestra is floored only by the splenial; (ii) the postparietal set is paired; (iii) an anterior process of parietal extends to midline of orbit; and (iv) orbital margin of jugal is somewhat V-shaped rather than rounded.

*Kuwavaatakdectes* shows a mix of character states that are plesiomorphic for Diadectomorpha, yet also possesses some more derived features. The lower jaw of MCZ 2989 is relatively shallow, so that the maximum depth does not exceed one-third of the jaw length. This is similar to most diadectids other than *D. sideropelicus* and *D. tenuitectus*. Its anterior incisiform teeth are procumbent in the lower jaw only, a primitive character state that it shares with other early-branching diadectids as *O. pabsti* but not any *Diadectes* species. Further, the cheek teeth are relatively simple with a single cusp like *D. absitus* and *D. dreigleichenensis*, but unlike the broadly expanded molariform teeth in *D. hesperis* and *D. sideropelicus*. The cheek teeth extend dorsally beyond the low labial parapet, as in most diadectids but the derived *D. sideropelicus*, *D. tenuitectus* and *D. zenos*. The pineal foramen is positioned at roughly the midlength of the interparietal suture (contra [40]) like *D. sideropelicus*, but unlike *O. pabsti* where it is clearly placed posteriorly. Also in contrast to *O. pabsti* but like other diadectids, the anterior margin of the otic embayment is near vertical for most of its length.

*Remarks*: Lewis & Vaughn [89] listed three diagnostic features for ‘*D*.’ *sanmiguelensis* to distinguish it from other members of *Diadectes*: (i) a shallow lower jaw whereby the maximum depth, at the coronoid, does not exceed a third of the jaw length; (ii) a shallow labial parapet; and (iii) the simple pattern of the cheek teeth with only a single cusp. However, this diagnosis would not differentiate *‘D.’ sangmiguelensis* from *D. absitus*. Lewis & Vaughn [89] nonetheless noted various diagnostic cranial features for the species, some of which scored in our character matrix, but they did not include those in the diagnosis. In his doctoral thesis, Kissel [40] added the splenial solely flooring the Meckelian fenestra as an autapomorphy of ‘*D*.’ *sanmiguelensis*, and further referred to the description of Lewis and Vaughn. Therein, he erected the new genus, *‘Oradectes’*, which has to be considered a *nomen nudum* under ICZN Article 13 and is thus unavailable. Lastly, Kissel [40] corrected one error in the description of Lewis & Vaughn [89]. Lewis and Vaughn indicated the presence of a separately ossified intertemporal between the right postfrontal and parietal, which would be a diagnostic feature, but Kissel [40] interprets this structure as part of the parietal lappets. We confirm the observations of Kissel [40].

*Etymology*: Ute, Kuwavaatak = edge; Latin, -*dectes* = biter. Kissel [40] proposed the generic name *Oradectes* (Latin, *ora* = margin) after the splenial representing the sole contributor to the ventral margin of the Meckelian fenestra, an apomorphy of the species. As Kissel’s [40] PhD thesis was not formally published, the generic name *Oradectes* has to be considered a *nomen nudum*. Though in the same spirit, the name Kuwavaatakdectes follows Kissel’s [40] suggestion, yet also honouring the Ute language of the Núuchi-u people that are native to Colorado.

## 4. Discussion

### 4.1. Implications of microcomputed tomography data for phylogenetic studies

Using µCT imaging techniques, we updated and expanded the previous character matrix of Clack *et al.* [37] with otherwise obscured characters in the lower jaw. Recent studies highlighted the potential of µCT data in phylogenetic studies on Palaeozoic tetrapods using endocranial characters like brain case anatomy [38,39,61,90–93] and cranial innervation [94,95]. We expanded on the subset of characters related to the mandible, increasing from 42 out of 294 (14.3%) in the previous iterations [37,39] to 69 out of 341 characters (20.2%). Scoring of these mandibular characters is impeded by the occlusion with the cranium in many Palaeozoic tetrapods, especially when that represents the only known specimen. This is reflected in the low count of OTUs for which certain characters could be scored; for example, the geometry of the tooth row (Ch. 322, 20 out of 61; 32.7%) or the anatomy of the glenoid surface (Ch. 328, 21 out of 61; 34.4%). Further µCT scanning of skulls of Palaeozoic tetrapods could thus vastly improve our knowledge of the variation in lower jaw anatomy and its phylogenetic significance.

It should however be noted that the density of the rock (especially when rich in iron and heavy metals) in occluded skulls occasionally reduces the resolution towards the centre, and obscures for instance in the palatal and postcoronoid regions. The variation in resolution throughout the scan was especially apparent in the skull of MNG 14473. Moreover, finer details like the minute fields of denticles on palatal and jaw bones reported in diadectomorphs [45,60] could not be discerned in our scans.

### 4.2. Phylogenetic position of Diadectomorpha

With the exception of Diadectomorpha, all clades containing Late Carboniferous–early Permian herbivorous tetrapods are unambiguously classified as members of Amniota [33–36] (but see Simões *et al.* [96] for a different interpretation on the position of Captorhinidae outside of crown-amniotes). However, the phylogeny of Diadectomorpha is particularly contentious. Several studies supported the traditional stem-amniote hypothesis [33–35,40,97], whereas others hypothesized a synapsid-placement [37,39,48,61,98]. Even the monophyly of Diadectomorpha has been challenged [99,100]. The extensive work on Palaeozoic tetrapod phylogeny by Marjanović & Laurin [101] moreover highlights that the monophyly, placement and content of Diadectomorpha vary with different analyses performed on the same character–taxon dataset; diadectomorphs were sometimes found paraphyletic with respect to Sauropsida, and the position of *Limnoscelis* was highly volatile. In our analysis, both the majority-rule consensus and Adams consensus recover a monophyletic Diadectomorpha including Limnoscelidae (figure 12). Our topology of Diadectomorpha closely follows that of Kissel [40]. In contrast to Kissel [40], we recover *D. tenuitectus* as the sister-taxon to *D. absitus* and *D. dreigleichenensis* rather than in a polytomy with *D. sideropelicus* and *D. zenos*. Moreover, in our majority-rule consensus, Diadectomorpha is retained within Amniota, more closely related to synapsids than to sauropsids (figure 12*a*).

Additionally, we were able to test the phylogenetic position of two purported diadectids, *A. pusillus* [80] and *A. fenestratus* [81], two taxa that have not been previously included in broad-scale phylogenetic studies beyond Diadectomorpha.

Kissel & Reisz [80] described *A. pusillus* as an early-branching diadectid. In their phylogeny, they recover *A. pusillus* as closer to *Diadectes* spp. than *T. campi* and *L. paludis*, but more basal than *O. pabsti*. This was repeated in Kissel [40], in which *A. pusillus* was found to be closer to *Diadectes* spp. than *T. campi* but more basal than *K. sanmiguelensis* and *O. pabsti,* respectively. Recently, Bulanov [102] questioned the diadectid assignment of *A. pusillus* and suggested a bolosaurian affinity instead. Currently, *A. pusillus* is known only from isolated maxillae and dentaries [80], so few characters could be scored. One character that supports an affinity with diadectids is the posterodorsal process on the dentary. Our majority-rule consensus tree recovers *A. pusillus* as a diadectid (figure 12*a*), conforming with Kissel & Reisz [80] and Kissel [40]. On the other hand, the Adams consensus tree places *A. pusillus* outside of Amniota + Diadectomorpha (figure 12*b*). Nevertheless, our analysis did not include any bolosaurian parareptile to test the hypothesis of Bulanov [102].

None of our consensus topologies support the position of *A. fenestratus* as a diadectid (figure 12)—contra Liu & Bever [81]. One character that Liu & Bever [81] cite in favour of a diadectid placement is the presence of a ‘sigmoid’ tooth row. While the tooth row indeed does appear sigmoidal in the reconstruction of the lower jaw in occlusal view according to Liu & Bever [81], the tooth row neatly follows the sigmoid lingual margin of the jaw. It does not therefore possess the ‘sinusoid’ tooth row geometry, meandering from the labial to lingual side unique to the Bromacker diadectids. Other diadectid characters such as a posterodorsal process of the dentary and an anterior blade-like process of the articular are not discernible in *A. fenestratus*. Instead, we find this taxon in a polytomy with the eothyridid synapsids (figure 12*a,b*). This placement is closer to the suggestion of Spindler *et al.* [103] that *A. fenestratus* represents a therapsid, which is based on the presence of dentary fangs. Our dataset does not contain any therapsid taxa to test this hypothesis. Nevertheless, this finding removes the need of a 46 Ma ghost lineage of diadectids that survived into the late Permian [81].

Lastly, the placement of *Varanops* is noteworthy. Varanopidae has generally been interpreted as an early-branching synapsid clade [95,96,104,105]. However, Ford & Benson [106] recently recovered the clade within Neodiapsida. We find *Varanops* robustly as a synapsid, in all three topologies (figure 12), as the sister-taxon to *Dimetrodon*. This finding is in agreement with the previous iterations of the character matrix used in this study [37,39,61].

In the recent phylogenetic analysis of Simões *et al.* [96], which focuses on more derived amniote groups, both Captorhinidae and Diadectomorpha are recovered as stem-amniotes. Captorhinidae forms a clade with Araeoscelidia as the sister-group to Amniota. Strikingly, this Captorhinidae–Araeoscelidia clade contains all taxa that Klembara *et al.* [61] refer to as ‘Sauropsida’. If we thus follow the hypothesis of Simões *et al.* [96], and Captorhinidae–Araeoscelidia falls outside of Amniota, the results of the studies by Klembara *et al.* [39] and Clack *et al.* [37] do not imply a synapsid, nor an amniote classification for Diadectomorpha—it would merely indicate that Diadectomorpha are closer to the crown-amniote group than Captorhinidae–Araeoscelidia.

The paucity of unambiguous amniotes in the current matrix, in addition to the relatively low support values overall, refrains us from making definitive statements regarding the precise placement of Diadectomorpha with respect to Amniota. Future studies should aim to include more amniotes in this character–taxon matrix.

### 4.3. Costal respiration, a key step towards herbivory?

Our results indicate herbivory in tetrapods is limited to amniotes (or, at the very least, to amniotes and their direct sister-group). This suggests that a key adaptation required for tetrapod herbivory has evolved only in this group. One compelling key adaptation that allowed amniotes to become herbivorous was put forward by Janis & Keller [107]. These authors suggested that a shift in the primary mode of respiration from buccal pumping in non-amniote tetrapods to costal respiration in amniotes relieved a constraint on skull anatomy and allowed for its diversification into forms suitable for herbivory. Buccal pumping is the ancestral mode of respiration for tetrapods [108–111], involving expansion and contraction of the buccal cavity to pump air into the lungs under positive pressure. Costal respiration, on the other hand, involves the expansion and contraction of the rib cage to fill the lungs. Non-amniote tetrapods plesiomorphically possess short, immobile ribs that are incapable of this movement [107]. All extant amniotes are capable of costal respiration [108,109], yet an advanced form of buccal pumping alongside costal respiration is retained in some lepidosaurs [111,112]. Thus, costal respiration likely evolved as an alternative to buccal pumping along the lineage leading to Amniota and functions as the primary mode of respiration in most taxa.

Janis & Keller [107] hypothesized that buccal pumping puts a constraint on skull shape, as this type of respiration would favour a skull that is broad compared with its length and dorsoventrally flattened, as a pair of bellows. This skull shape is poorly suited for herbivory. Using a dataset of 133 non-amniote tetrapod and 97 amniote taxa, encompassing both extant and extinct representatives, Janis & Keller [107] showed that non-amniote tetrapod skulls are consistently broader and are more limited in their head-to-body ratio than those of amniotes. Moreover, the skull shape of extinct non-amniote tetrapods more closely resembles that of extant amphibians than of extant amniotes. The data presented by Janis & Keller [107] thus support the hypothesis that buccal pumping constrains skull shape. However, it should be noted that a shift to costal respiration does not necessitate change to skull shape, but rather allows for skull shape to change. Nonetheless, the subsequent development of a deeper skull and associated restructuring of jaw adductor musculature in amniotes is interpreted to have been necessary for the evolution of herbivory [107,113]. Specifically, the insertion of the pterygoideus muscle to a fully developed pterygoid flange in amniotes would allow for static pressure, i.e. the ability to exert force when the mandible is in occlusion. This is required when applying a force with the front of the mandible with a closed mouth, for example, to crop vegetation with the anterior chisel-like teeth in diadectids. Moreover, the restructuring of jaw-closing musculature in amniotes, by extension, would allow the mandible to diversify as well. Indeed, the advent of amniotes and diadectomorphs greatly expanded the mandibular functional morphospace of tetrapods [10].

However, it is difficult to infer whether diadectomorphs and early amniotes were capable of costal aspiration or to pinpoint at which evolutionary grade this mode of breathing evolved. Ventilatory action in amniotes involves the sternum and sternal ribs [109], which are not known to ossify in early amniotes nor diadectomorphs. Janis & Keller [107] nevertheless proposed a list of osteological correlates to infer a mobile ribcage articulating with a cartilaginous ventral structure connecting to a similarly cartilaginous sternum, which would indicate that costal respiration is at least possible. These include a mobile vertebra–rib joint, rib heads with well-separated capitulum and tuberculum, as well as non-overlapping long, mesiodistally curved ribs with distal articulatory surfaces. This morphology is in stark contrast to the generally short and immobile ribs, whereby the capitulum and tuberculum remain merged, as seen in non-amniote tetrapods such as lissamphibians [114] and temnospondyls [99]. A bicapitate rib morphology is widely spread among amniotes [115] and likely represents the ancestral condition for this clade [109]. While a web of bone still connects the capitulum and tuberculum, Janis & Keller [107] confirm a bicapitate rib in *D. tenuitectus*. Moreover, similar costal anatomy has been described in other diadectomorphs as *L. paludis* [46], *T. campi* [49], *O. pabsti* [56] and *D. absitus* [60], early synapsids as *Ophiacodon uniformis* [107] and *M. bromackerensis* [57], as well as early diapsids like *P. kansensis* [116]. Thus, anatomy consistent with costal respiration was thus likely present at the common ancestor of amniotes and diadectomorphs. This innovation could therefore have allowed the skull to diversify and repeatedly develop adaptations to herbivory within Amniota and diadectomorphs. Future studies should consider the evolution of costal anatomy along the amniote stem-group in more detail, in conjunction with the offset of morphological diversification of both the cranium and the lower jaw, to test this hypothesis further.

### 4.4. The diverse herbivore fauna at Bromacker

The Bromacker locality is unique among early Permian localities for preserving a herbivore-dominated fauna, reflecting a modern ecosystem [52]. Four herbivorous taxa have previously been described: the bolosaurid *E. cursoris* [58], the caseid *M. bromackerensis* [57], as well as the diadectids *D. absitus* and *O. pabsti* [56,60].

*Eudibamus* is interpreted as an agile animal that was capable of bipedal locomotion based on the well-preserved postcranial material [58]. Like other bolosaurids [2], *Eudibamus* would have been a pickier herbivore scurrying for low-fibrous yet more nutritious young leaves and would take small prey if opportunistically encountered.

*Martensius* is the sole representative of Caseidae at Bromacker, and fits in a similar size class as the co-occurring diadectids. Its ribcage is not as widely expanded like in more derived caseids such as *Cotylorhynchus romeri* [117], but is representative of earlier and basal caseasaurs. The simple isodont dentition does not support powerful mastication. Instead, the disproportionally large front limbs packed with strongly recurved bony claw unguals indicate *Martensius* was potentially able to dig for plant food [57], as suggested for other caseids [2,117].

The diadectid fauna at Bromacker is more taxonomically diverse than previously appreciated. *Orobates* differs from *Diadectes* in several aspects, notably by the presence of a shallow lower jaw, spatulate anterior teeth and lesser degree of molarized cheek teeth and a short anterior process of the articular [56]. These characters, in combination with an uneven wear pattern on the cheek teeth in MNG 11134 favouring a primarily orthal jaw motion, led Berman *et al.* [56] to conclude that *Orobates* was less adapted to processing high-fibre foods than *Diadectes*.

The addition of the new taxon *D. dreigleichenensis* proposed in this paper adds to the already extensive diversity of both herbivorous and diadectid taxa at the locality. The full list of differences in skull anatomy between *D. absitus* MNG 8853 and *D. dreigleichenensis* MNG 8747 compiled from Berman *et al.* [60] and this study are listed in table 1. Finally, anatomical variation in the lower jaw suggests a different way of food processing between the two *Diadectes* species at Bromacker. While the mandible of MNG 8747 is less deep than those of *D. absitus*, it possesses a strongly interdigitated sutural contact of the angular and prearticular at the level of the apex of the coronoid eminence. Interdigitated sutures form as a result of tension, perpendicular to main vectors of stress [118]. This would indicate a tendency to an orthal jaw movement and perhaps a preference for tougher food items than *D. absitus*, suggesting the possibility of niche partitioning between the rich herbivore fauna. The Bromacker palaeoenvironment is thus even more diverse and ecologically complex than previously assumed.

## 5. Conclusions

We provide the most comprehensive phylogenetic analysis of the inter- and intrarelationships of Diadectomorpha to date, including all purported diadectomorph taxa known from sufficient material, and greatly expand the number of characters using new information on the lower jaw. Diadectomorpha are recovered as a monophyletic clade including *Limnoscelis* in both the majority-rule and Adams consensus trees. All three consensus trees further agree on a topology of derived diadectids. Following a detailed anatomical comparison between *D. absitus* holotype MNG 8853 and former paratype MNG 8747 from the early Permian Bromacker locality in Thuringia, Germany, the erection of a new species *D. dreigleichenensis* for MNG 8747 is warranted. Additionally, we erected the new genus *Kuwavaatakdectes* to accommodate *K. sanmiguelensis*. Furthermore, Diadectomorpha are found as the sister-group to Synapsida within Amniota in the majority-rule consensus tree, lending support to the findings of earlier iterations of this character matrix. Diadectomorpha contain the oldest known herbivorous tetrapods, and its position among Amniota would suggest that tetrapod herbivory is limited to amniotes. These new phylogenetic results, as well as their implications for the evolution of tetrapod herbivory, highlight the potential for additional research on the fascinating clade Diadectomorpha.

## Data Availability

All raw scan data are provided on MorphoSource repository project ID: 000594326. This article is registered in ZooBank (urn:lsid:zoobank.org:pub:6EB74A26-57C0-4B3E-A0E0-486F21223330). Supplementary material is available online [119].
